# Age-dependent alterations of monocyte subsets and monocyte-related chemokine pathways in healthy adults

**DOI:** 10.1186/1471-2172-11-30

**Published:** 2010-06-21

**Authors:** Sebastian Seidler, Henning W Zimmermann, Matthias Bartneck, Christian Trautwein, Frank Tacke

**Affiliations:** 1 Department of Medicine III, University Hospital, RWTH-Aachen, Pauwelsstr 30, 52074 Aachen, Germany; 2 Interdisciplinary Centre for Clinical Research, University Hospital, RWTH-Aachen, Pauwelsstr 30, 52074 Aachen, Germany

## Abstract

**Background:**

Recent experimental approaches have unraveled essential migratory and functional differences of monocyte subpopulations in mice. In order to possibly translate these findings into human physiology and pathophysiology, human monocyte subsets need to be carefully revisited in health and disease. In analogy to murine studies, we hypothesized that human monocyte subsets dynamically change during ageing, potentially influencing their functionality and contributing to immunosenescence.

**Results:**

Circulating monocyte subsets, surface marker and chemokine receptor expression were analyzed in 181 healthy volunteers (median age 42, range 18-88). Unlike the unaffected total leukocyte or total monocyte counts, non-classical CD14^+^CD16^+ ^monocytes significantly increased with age, but displayed reduced HLA-DR and CX_3_CR1 surface expression in the elderly. Classical CD14^++^CD16^- ^monocyte counts did not vary dependent on age. Serum MCP-1 (CCL2), but not MIP1α (CCL3), MIP1β (CCL4) or fractalkine (CX_3_CL1) concentrations increased with age. Monocyte-derived macrophages from old or young individuals did not differ with respect to cytokine release *in vitro *at steady state or upon LPS stimulation.

**Conclusions:**

Our study demonstrates dynamic changes of circulating monocytes during ageing in humans. The expansion of the non-classical CD14^+^CD16^+ ^subtype, alterations of surface protein and chemokine receptor expression as well as circulating monocyte-related chemokines possibly contribute to the preserved functionality of the monocyte pool throughout adulthood.

## Background

Monocytes represent about 5-10% of peripheral blood leukocytes in humans and mice. They originate from a myeloid precursor in the bone marrow, circulate in the blood, bone marrow and spleen, and then enter tissues [1,2]. Monocytes are established circulating precursors for tissue macrophages and dendritic cells (DCs). Migration of monocytes into tissues and differentiation into macrophages or DCs is believed to be largely determined by the inflammatory milieu, i.e. adhesion molecules, chemokines and pathogen-associated pattern-recognition receptors [1]. The identification of different monocyte subsets in mice [3] has prompted intensive research over the last years to understand the true contribution of monocyte subpopulations to the macrophage and tissue DC pool in inflammatory disorders, but also in steady state [1].

Heterogeneity among human monocytes was recognized about thirty years ago [4,5], and several markers like CD64 [6] or CD16 [7] have been suggested for differentiating subpopulations of monocytes. The differential expression of CD14 (part of the receptor for lipopolysaccharide) and CD16 (also known as FcγRIII) are commonly used to define two major subsets in peripheral blood: 'classical' CD14^++^CD16^- ^monocytes, typically representing up to 95% of the monocytes in a healthy individual, and the 'non-classical' CD14^+^CD16^+ ^cells comprising the remaining fraction of monocytes [7]. These subsets differ in many respects, including adhesion molecule and chemokine receptor (CCR) expression. CD14^++^CD16^- ^monocytes express CCR2, CD62L (L-Selectin) and FCγRI (CD64), whereas CD14^+^CD16^+ ^monocytes lack CCR2, and have higher levels of MHC-II and FCγRII (CD32). Both subsets express the receptor for fractalkine, CX_3_CR1, but CD14^+^CD16^+ ^monocytes characteristically express higher levels [2,8]. Based on similar adhesion molecule and chemokine receptor as well as similar gene expression profiles, murine Gr1^hi ^(Ly6C^hi^) monocytes are considered counterparts of human CD14^++^CD16^- ^monocytes, and murine Gr1^lo ^(Ly6C^lo^) cells may represent the subpopulation comparable to human CD14^+^CD16^+ ^monocytes [8].

Intensive research efforts have unraveled important functional characteristics of both monocyte subsets in mice. While the Gr1^hi ^monocytes are rapidly recruited to sites of inflammation, such as in atherosclerosis, peritonitis or after organ damage into the injured liver [9-12], Gr1^lo ^monocytes appear to have a more patrolling behavior at the endothelium [13]. Consequently, Gr1^hi ^monocytes were found to give rise to pro-inflammatory macrophages and TNF-producing DCs in inflamed tissue [1,9,10,14], while Gr1^lo ^monocytes have been proposed as precursors for alternatively activated macrophages, possibly fulfilling functions in tissue repair and resident macrophage/DC turnover [1,13,15]. All these studies have raised the question how this knowledge can be translated into understanding physiology and pathophysiology of monocyte subsets in humans. We therefore aimed at characterizing peripheral blood monocyte subsets in healthy volunteers based on frequency, phenotype, chemokine and chemokine receptor expression and functionality assays *ex vivo*. Data from mice [2] and humans [16] indicated that monocyte subsets considerably change with age. Here, we show that the 'non-classical' CD14^+^CD16^+ ^monocytes specifically increase with age in healthy volunteers, that monocyte-related chemokines and chemokine receptors are regulated within individuals of different ages and that the peripheral monocyte population preserves its cytokine-secreting function during ageing in adults.

## Methods

### Study participants

Healthy volunteers were recruited from the blood transfusion institute and from the staff or former staff of the Department of Medicine III of the University Hospital Aachen, Germany (Table 1). The study protocol was approved by the local ethics committee (ethics committee of University Hospital Aachen, RWTH Aachen), and written informed consent was obtained from each participant. A brief medical history was performed to exclude possible diseases. Besides the experimental analyses from peripheral blood, all participants were routinely tested for an automated blood count and differential blood count, normal alanine aminotransferase activity and normal C-reactive protein at the Institute for Clinical Chemistry of the University Hospital Aachen, Germany. Using these efforts, all participants, including the persons above 65 years of age, could be considered "healthy" based on admission criteria of the extensively validated 'senieur protocol' [17,18]. However, it is important that atherosclerosis cannot be ruled out following these criteria.

**Table 1:** Characteristics of the study cohort and experimental measurements.

		all volunteers	age groups [years]		
			<30	30 - 50	>50
	[n]	**181**	51	70	60

male/female	[n]	**105/76**	28/23	42/28	35/25

age	[years]	**42 (18-88)**	25 (18-29)	43 (30-50)	58 (51-88)

hemoglobin concentration	[g/L]	**139 (96-172)**	144 (122-172)	138 (96-162)	138 (104-159)

platelet count	[×10^9^/L]	**261 (150-509)**	275 (184-424)	265 (193-509)	252 (150-370)

total leukocytes	[×10^9^/L]	**5.9 (1.7-11.6)**	5.9 (3.6-11.6)	6.1 (1.7-10.2)	5.8 (3.3-9.5)

total monocytes	[×10^6^/L]	**450 (170-990)**	424 (170-810)	462 (170-990)	461 (190-880)

CD14^++^CD16^- ^monocytes	[%]	**7.05 (2.82-11.99)**	6.69 (2.82-11.49)	7.14 (2.86-11.93)	7.26 (3.44-11.99)

CD14^++^CD16^- ^monocytes	[×10^6^/L]	**412.8 (155.1-903.0)**	394.3 (155.1-776.5)	414.3 (165.7-902.9)	420.3 (165.2-902.9)

CD14^+^CD16^+ ^monocytes	[%]	**0.54 (0.14-1.93)**	0.42 (0.15-1.51)	0.59 (0.14-1.71)	0.69 (0.27-1.93)

CD14^+^CD16^+ ^monocytes	[×10^6^/L]	**33.7 (8.15-107.6)**	24.3 (8.15-107.6)	35.7 (8.28-106.1)	37.8 (14.8-106.3)

serum MCP-1/CCL2	[pg/mL]	**555 (61-1851)**	450 (109-880)	573 (122-1851)	654 (61-1563)

serum MIP1α/CCL3	[pg/mL]	**178 (14-10084)**	470 (14-10084)	172 (14-10000)	123 (14-10000)

serum MIP1β/CCL4	[pg/mL]	**32 (6-1107)**	27 (10-1107)	33 (6-413)	32 (11-200)

serum fractalkine/CX_3_CL1	[pg/mL]	**27.0 (0-80.5)**	22.4 (0-62.6)	30.5 (0-68.7)	27.0 (0-80.5)

The median and range (in parenthesis) is given for quantitative parameters. % refers to % of leukocytes.

### Isolation of PBMC and flow cytometry

Fresh blood samples were collected by venipuncture in the morning in EDTA separator tubes and promptly applied to PBMC isolation by Ficoll Density Gradient, using LSM 1077 Lymphocyte Separation Medium (PAA, Pasching, Austria) and centrifugation at 2200 rpm for 20 minutes at 20°C. The intermediate layer consisting of peripheral blood mononuclear cells (PBMC) was washed twice in HANKS's medium (PAA) containing 0.1% BSA and 0.5 mM EDTA and twice with DMEM Buffer (PAA) containing 2 mM EDTA and 0.5% BSA [8,19]. After blocking of nonspecific antibody binding, the following monoclonal antibodies and appropriate isotype controls were used for flow cytometry: CD14, CD16, CD56, HLA-DR, CD3, CD4, CD8, CD56, and CD19 (all BD); CCR2 (R&D Systems); CX_3_CR1 (MBL International, Woburn, MA). Flow cytometric analysis was performed on a FACS Canto-II (BD). The acquired data were analysed by FlowJo software (TreeStar, Ashland, OR). Numbers of circulating cells were assessed by the percentage of the respective cell subset multiplied by the respective subset of absolute cell count obtained from routine blood count. Cell surface marker expression was quantified by determining median fluorescence intensity minus the respective isotype control from an otherwise fully stained sample (MFI-FMO). Due to technical reasons, HLA-DR, CCR2 and CX_3_CR1 stainings have only been performed on 130/181 and 62/181 consecutively included subjects, respectively.

### Chemokine and cytokine detection

The release of chemokines/cytokines in human serum or in culture medium supernatant was measured using FlowCytomix (Bender Medsystems, Austria, Vienna) in collaboration with the company, according to manufacturers' instructions. The system of cytokine detection is based on antibody-coupled micro-bead populations of which each specifically binds to a certain cytokine. The antibody-coupled microspheres serve as a solid phase in this sandwich immunoassay methodology, and multiple sphere populations can be distinguished on a flowcytometer and each population is coated with a different antibody. Measurements were performed in duplicates at 50 μL sample volume. Standards and samples were distributed in 96-well plates. Mixture of coated beads and detection beads was added and incubated for one hour. After washing twice Streptavidin-PE was added and incubated for one hour. After washing twice the measurements were done using a flowcytometer and analyzed by FlowCytomixPro software (Bender) [19]. Serum concentrations of Fractalkine were assessed by Cytometric Bead Assay (BD, Heidelberg, Germany) according to manufacturer's instructions [9].

### Purification of monocytes, generation of monocyte-derived macrophages and in-vitro stimulation

For isolation of peripheral monocytes blood was freshly drawn from healthy volunteers in fully heparinised syringes and, after diluting with PBS at a ratio of 1:1, directly subjected to Ficoll isolation using LSM 1077 Lymphocyte Separation Medium (PAA, Pasching, Austria) and centrifugation at 1600 rpm for 40 minutes at room temperature. PBMC were washed using PBS until the supernatant was clear. 1 × 10^6 ^cells/ml were resolved in 4 ml 1640 RPMI (Invitrogen) containing 1% penicillin-streptomycin (PAA) and 1.5% heat inactivated autologous serum and allowed to adhere for 35 min in Petri dishes (Greiner Bio-one, Frickenhausen, Germany) at 37°C and 5% CO_2_. Non-adherent cells were discarded and the adherent cell layer was washed five times with 37°C warm 1640 RPMI solution. The cells were then resolved in 2 ml RPMI (5% heat inactivated autologous serum, 1% Penicillin-Streptomycin). After 24 h in culture, cells were either stimulated with 1 μg/mL LPS (Sigma-Adrich, Hamburg, Germany) or left unstimulated. After further incubation for 24 hours, supernatants were collected and stored at -80°C.

### Statistics

Data are given as median, minimum, maximum, and shown graphically by box-and-whiskers plots [20]. The box-and-whiskers plots display a statistical summary of the median (bold line), quartiles (boxes), range and extreme values. The whiskers extend from the minimum to the maximum value excluding outside (>1.5 times upper/lower quartile, open circle) and "far out" (>3 time upper/lower quartile, asterixes) values which are displayed separately. The degree of association between two variables was assessed by the Spearman rank correlation test. Comparisons of parameters between two different groups were conducted with the Mann-Whitney-U-test. Comparisons between three groups (e.g., volunteers of different age classes) were done with the Kruskal-Wallis analysis of variances (ANOVA), followed by Mann-Whitney-U-tests for post hoc analysis. All tests were carried out with SPSS (SPSS, Chicago, IL), and p-values < 0.05 were considered statistical significant.

For the *in vitro *experiments, bar graphs represent the mean and the standard error of the mean (SEM). Statistical comparisons between groups were performed using the Mann-Whitney-*U*-test with Prism 3.0 (GraphPad). P-values < 0.05 were considered statistical significant.

## Results

### The non-classical CD14^+^CD16^+ ^monocyte subset specifically increases with age in healthy adults

**Figure 1:**
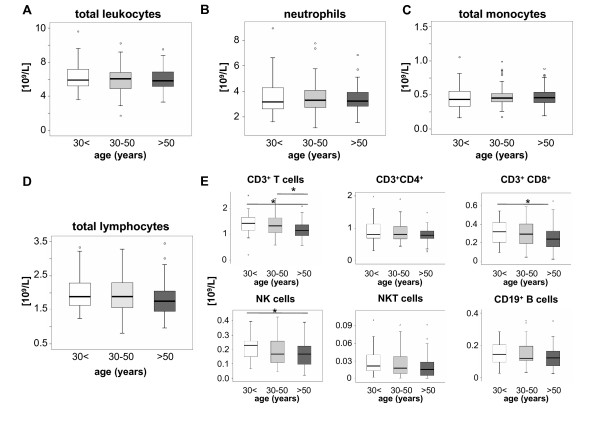
**Age-dependent changes of circulating leukocytes**. Box plots display absolute numbers of circulating total leukocytes (A), neutrophils (B), monocytes (C), lymphocytes (D) and lymphocyte subsets (E) in 10^9^/L for the different groups of young (<30 years, n = 51), middle-aged (30-50 years, n = 70) and old (>50 years, n = 60) healthy volunteers. The bold black line indicates the median per group, the box represents 50% of the values, and horizontal lines show minimum and maximum values of the calculated non-outlier values; open circles indicate outlier values. Significant differences (Kruskal-Wallis and U-test) are marked by *p < 0.05.

We studied circulating leukocyte subsets from 181 healthy volunteers at different ages by subjecting fresh PBMC to immediate FACS analysis (Table 1). Within healthy adults, total numbers of circulating white blood cells, neutrophils or total monocytes did not change dependent on the age of the volunteers (Fig. 1A-C). Moreover, no differences were observed between male and female volunteers (data not shown). Within the lymphocytes (Fig. 1D-E), a slight decrease in circulating T cells, identified by positive staining for CD3 and negativity for CD56, was noted in persons above 50 years of age that could be attributed to lower numbers of CD8^+ ^T cells (Fig. 1E). A similar observation was found for circulating NK cells (CD56^+^CD3^- ^cells) that were significantly reduced in volunteers above 50 years (Fig. 1E).

**Figure 2:**
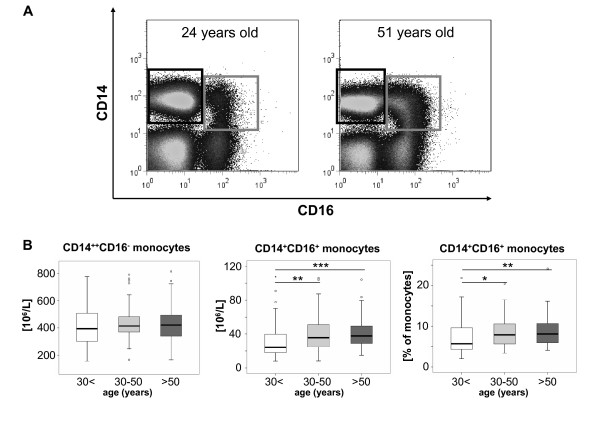
**Age-dependent changes of circulating monocyte subsets**. (A) Representative FACS plots display an increase of CD14^+^CD16^+ ^monocytes (grey gate) in comparison to classical CD14^++^CD16^- ^monocytes (black gate) among freshly isolated PBMC from old (right plot, 51 years-old male) versus young (left plot, 24 years-old female) volunteers. (B) Box plots display absolute numbers of circulating CD14^++^CD16^- ^monocytes (left), CD14^+^CD16^+ ^monocytes (middle) and the relative percentage of CD14^+^CD16^+ ^from total monocytes (right) for the different groups of young (<30 years, n = 51), middle-aged (30-50 years, n = 70) and old (>50 years, n = 60) healthy volunteers. The bold black line indicates the median per group, the box represents 50% of the values, and horizontal lines show minimum and maximum values of the calculated non-outlier values; open circles indicate outlier values. Significant differences (Kruskal-Wallis and U-test) are marked by *p < 0.05, **p < 0.01 and ***p < 0.0015.

Circulating monocytes were further subdivided by FACS analysis into CD14^++^CD16^- ^and CD14^+^CD16^+ ^(also called CD14^dim^CD16^bright^) monocytes according to the expression of CD14 and CD16 on their surface (Fig. 2A). Although the number of total monocytes did not differ, we observed a clear correlation between the 'non-classical' CD14^+^CD16^+ ^monocyte subset and age (r = 0.238, p = 0.001, Spearman rank correlation test). Both the absolute numbers of the 'non-classical' CD14^+^CD16^+ ^monocytes as well as their relative contribution to the circulating monocyte pool increased with the age of the study participants (Fig. 2B). No difference was found between male and female individuals (data not shown).

### Age-dependent changes of monocytic chemokine receptor expression

**Figure 3:**
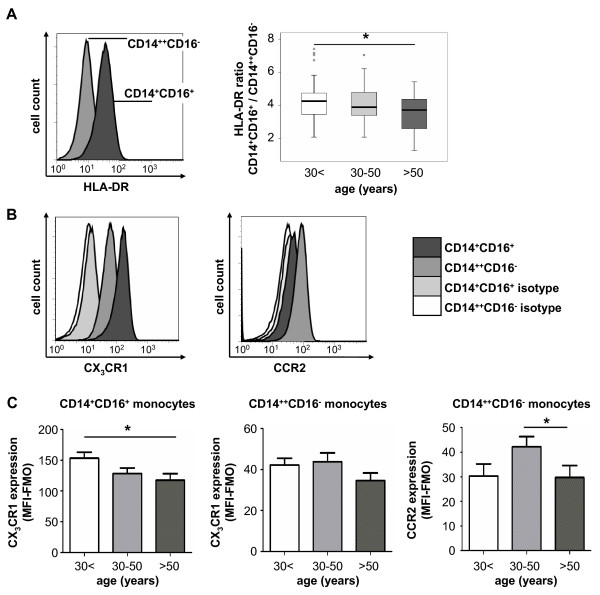
**Age-dependent changes of HLA-DR and chemokine receptor expression on monocyte subsets**. (A) HLA-DR expression was analyzed by FACS on monocyte subsets (representative histogram of a 52-years old male is shown). Box plots demonstrate alterations of HLA-DR expression on both subsets using the ratio of HLA-DR between CD14^+^CD16^+ ^and CD14^++^CD16^- ^monocytes for the different groups of young (<30 years, n = 37), middle-aged (30-50 years, n = 50) and old (>50 years, n = 43) healthy volunteers. *p < 0.05. (B) Chemokine receptor expression was studied by FACS on both circulating monocyte subsets. Representative histograms (from a 65-years old male) display that CD14^+^CD16^+ ^monocytes express higher levels of CX_3_CR1, whereas CCR2 is primarily detected on classical CD14^++^CD16^- ^monocytes. (C) Bar graphs depict changes in chemokine receptor expression (quantified as median fluorescence intensity minus "fluorescence minus one", MFI-FMO) for both monocyte subsets for the different groups of young (<30 years, n = 21), middle-aged (30-50 years, n = 22) and old (>50 years, n = 19) healthy volunteers. *p < 0.05.

We next aimed at identifying phenotypic differences between monocyte subsets of young and old(er) adults. We therefore analyzed the expression of the MHC-II molecule HLA-DR that is considered as an activation marker of monocytes and found at higher levels on CD14^+^CD16^+ ^monocytes [2,8]. Interestingly, the expression of HLA-DR was significantly lower on CD14^+^CD16^+ ^monocytes of older volunteers, which was apparent by analyzing the ratio of HLA-DR expression between CD14^++ ^and CD14^+^CD16^+ ^monocytes as well as by directly analyzing HLA-DR levels on each subset (Fig. 3A and Additional file 1).

The chemokine receptors CCR2 and CX_3_CR1 are differentially expressed on both monocyte subsets and have been implicated in their migration and function [10]. While CD14^++^CD16^- ^monocytes express high levels of CCR2 and low levels of CX_3_CR1, CD14^+^CD16^+ ^monocytes express very low levels of CCR2 and high levels of CX_3_CR1 (Fig. 3B). Interestingly, expression of CX_3_CR1 was downregulated on CD14^+^CD16^+ ^monocytes in older volunteers compared to young adults (Fig. 3C), but not downregulated on CD14^++^CD16^- ^monocytes. The 'classical' monocytes displayed some variation in CCR2 expression, with highest levels in volunteers at the age between 30 and 50 years (Fig. 3C). Collectively, these data indicate that not only the absolute number of the 'non-classical' CD14^+^CD16^+ ^monocytes increase with age, but also their phenotype changes dependent on age, resulting in lower expression of activation markers and chemokine receptors.

### Serum MCP-1 concentrations, but not other monocyte-related chemokine levels, increase with age

**Figure 4:**
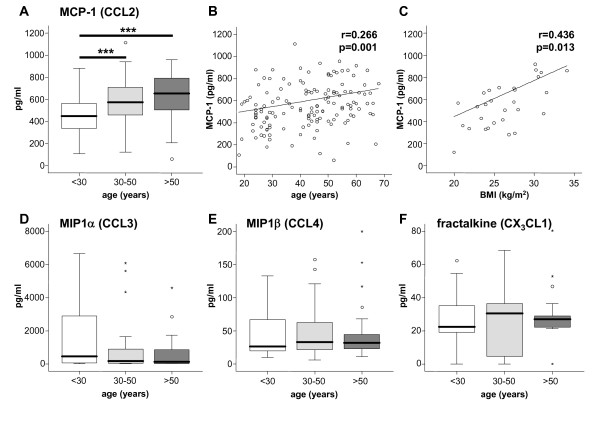
**Age-dependent changes of circulating monocyte-related chemokine levels**. Box plots display serum concentrations of the chemokines MCP-1 (A), MIP1α (D), MIP1β (E) and fractalkine (F) for the different groups of young (<30 years, n = 51), middle-aged (30-50 years, n = 70) and old (>50 years, n = 60) healthy volunteers. The bold black line indicates the median per group, the box represents 50% of the values, and horizontal lines show minimum and maximum values of the calculated non-outlier values; open circles indicate outlier values. Significant differences (Kruskal-Wallis and U-test) are marked by ***p < 0.0015. (B+C) MCP1 serum concentrations correlate with the age (r = 0.266, p = 0.001) and the body-mass index (BMI, r = 0.430, p = 0.013) in healthy volunteers (Spearman rank correlation test).

In various experimental animal models of inflammatory disorders, the chemokine receptors CCR2, CCR1, CCR5 and CX_3_CR1 are involved at different stages of monocyte subset migration [10,21]. Of note, MCP-1, the ligand for CCR2, regulates Gr1^hi^/Ly6C^hi ^'classical' monocyte migration by promoting their exit from the bone marrow into the circulation in mice [14,22]. In agreement with these observations from mice, we found increased systemic concentrations of MCP-1 in the serum of healthy volunteers dependent on their age (Fig. 4A-B). Moreover, the body-mass index positively correlated with serum MCP-1 levels (Fig. 4C). In contrast, circulating concentrations of MIP1α and MIP1β, both ligands of CCR1 and CCR5, or fractalkine, the ligand for CX_3_CR1, were not associated with the age of the volunteers (Fig. 4D-F).

### Monocyte-derived macrophages remain functionally active in healthy aged volunteers

**Figure 5:**
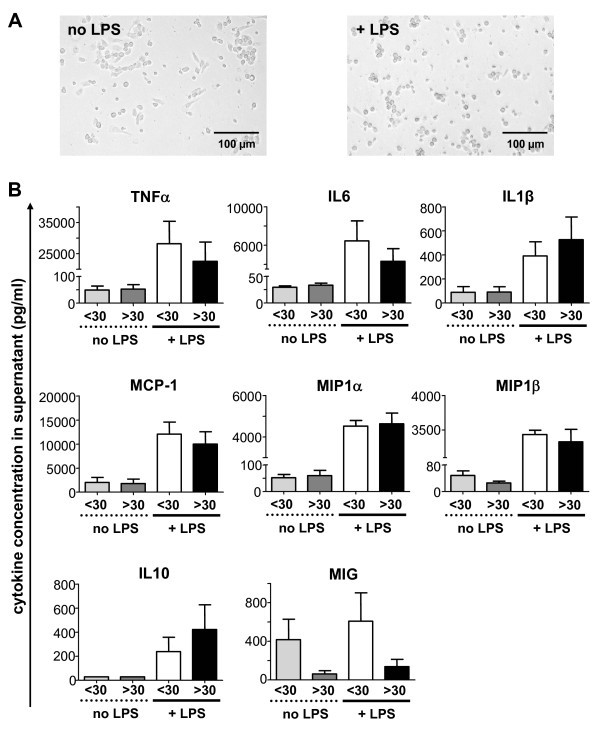
**Functional properties of monocyte-derived macrophages from young and old volunteers**. Macrophages derived from freshly isolated PBMC from n = 12 volunteers aged <30 years (<30) and from n = 10 volunteers aged >30 years (>30) were cultured in medium containing autologous serum for 24 h and then stimulated with 1 μg/ml LPS for additional 24 h or left untreated. (A) Macrophages display an activated, round-shaped morphology after LPS stimulation, but no overt morphological differences were detected between macrophages from young or old adults. (B) Bar graphs show mean cytokine and chemokine levels (in pg/mL) in the supernatant at day 2, error bars represent SEM.

In order to assess possible alterations of the functionality of monocytes dependent on the age, we tested the capacity of monocyte-derived macrophages to produce cytokines *in vitro*. An imbalanced cytokine production by monocyte-derived macrophages had been reported in a prior study of geriatric volunteers (with a mean age of 88 years) [16], but similar data for adults of different age groups are lacking. Monocytes were isolated from n = 12 young (median age 24, range 21-28) and n = 10 old adults (median age 60, range 35-88) and differentiated *in vitro *into macrophages by overnight culture in medium supplemented with autologous serum. We then assessed spontaneous cytokine/chemokine production as well as after stimulation with LPS. No morphological differences were visible between monocyte-derived macrophages from young or old volunteers (Fig. 5A). In line, constitutive cytokine/chemokine production did not differ dependent on the age (Fig. 5B). Monocyte-derived macrophages secreted high amounts of the proinflammatory cytokines TNFα, IL6, IL1β, of the antiinflammatory cytokine IL10 and also the chemokines MCP-1, MIP1α and MIP1β upon stimulation with LPS, while MIG was not significantly induced by LPS. Again, cells derived from old volunteers displayed a fully preserved functional capacity to produce these cytokines/chemokines upon LPS stimulation (Fig. 5B).

## Discussion

Alterations in the numbers, phenotype and functionality of monocyte subsets have recently gained remarkable attention, because the identification of murine monocyte subpopulations has revealed new insights into their critical functions for macrophage and dendritic cell homeostasis as well as during inflammatory responses. For instance, the appearance of monocyte subsets in peripheral murine blood is the result of several well regulated processes at the level of bone marrow exit, half-life in blood and release from the spleen that can serve as an important reservoir [1,14,23]. Their differential recruitment in steady state or to sites of inflammation involves a cascade of contacts with endothelial proteins, adhesion molecule expression and chemokine-chemokine receptor interactions [10,13,24], and some of these mechanisms are highly specific for each monocyte subset, resulting in their selective or sequential accumulation and determining their function in health and disease [1,9,10,15,24]. It is an ongoing challenge to establish which of these mechanisms can be translated into human physiology and pathophysiology.

We here conducted a study aiming at thoroughly characterizing alterations of monocyte subpopulations in healthy human volunteers at different ages. The effects of ageing on adaptive and innate immune responses in humans remain incompletely understood. The increased susceptibility to infections and the reduced response to vaccinations in old people have been linked to T- and B-cell functions [25], but also changes in the innate immune systems, including neutrophils, monocytes, macrophages, natural killer and natural killer T (NKT) cells and dendritic cells, have been reported to contribute to the "immunosenescence" observed at old age [26]. Our study demonstrates a significant shift from 'classical' CD14^++^CD16^- ^to 'non-classical' CD14^+^CD16^+ ^monocytes with increasing age in healthy adults. These results are well in agreement with a prior smaller study that reported a significant expansion of CD14^+^CD16^+ ^monocytes in the elderly, although both studies are not directly comparably, because the prior study compared very old (mean age 88 years) with young (mean age 30 years) subjects [16].

Moreover, the CD14^+^CD16^+ ^monocytes undergo phenotypic changes during ageing, as they display reduced levels of the activation molecule HLA-DR and of the key chemokine receptor CX_3_CR1. Especially the latter finding might be functionally relevant, because CX_3_CR1 has been implicated to promote monocyte/macrophage survival in some conditions like atherosclerosis [27]. Given experimental data from mice, reduced CX_3_CR1 expression on non-classical monocytes in humans is likely to affect their migration to inflammatory sites and to the spleen [10,28], but could also reduce the half-life of monocyte-derived tissue macrophages [29]. Therefore, increased numbers of circulating CD14^+^CD16^+ ^monocytes may not necessarily mean enhanced availability and functionality in aged persons.

At the level of circulating chemokines, we observed a marked increase of serum MCP-1 (CCL2) concentrations with age. Independent studies have reported increasing MCP-1 concentrations in older volunteers previously and speculated that this could be related to development of (subclinical) atherosclerosis or an age-dependent shift between T-helper cell dependent cytokine patterns (Th1/Th2) [30,31]. In our cohort, we observed a correlation between serum MCP-1 and the body mass index, possibly indicating an association with obesity and adipose tissue-derived macrophages or obesity-induced hepatic MCP-1 expression as important sources of circulating MCP-1 [32,33]. Interestingly, neither was CCR2 expression downregulated on CD14^++^CD16^- ^monocytes in response to MCP-1 levels nor did serum concentrations of fractalkine (CX_3_CL1) or the CCR1/CCR5 ligands CCL3 (MIP1α) and CCL4 (MIP1β) change with age.

Surprisingly, our experiments further indicate that the observed changes in the number and phenotype of circulating monocyte subsets do not largely impact their overall functionality, at least not in the studied age-range of healthy adults (mean age 41 years, 95%-interval 20-68 years). This is unexpected, because CD14^+^CD16^+ ^monocytes, which are significantly expanded in older volunteers, are generally believed to have a much higher capacity to secrete proinflammatory cytokines [2]. We recently confirmed these assumptions, as CD14^+^CD16^+ ^monocytes derived from healthy volunteers produce significantly more TNF, IL6, MIP1α, MIP1β, IFNγ and MIG upon culture without specific stimulation, while CD14^++^CD16^- ^monocytes readily release more MCP-1 and IL-10 *in vitro *[19].

In our study, the constitutive and inducible synthesis of cytokines and chemokines was similar between total monocytes cultured from young and old(er) adults, although the fraction of the CD14^+^CD16^+ ^subset was expanded in the cells derived from older persons. Possibly, the expansion of the 'non-classical' monocytes with age might represent a physiological counter-regulatory reaction, in order to preserve the overall functionality of the monocyte pool throughout life-time. However, it is important to note that our experimental setting did not allow to differentiate the distinct contribution of each subset to the overall cytokine release and that our *in-vitro *results do not necessarily have to reflect the true behaviour of monocyte-derived cells in their respective (inflamed or non-inflamed) *in-vivo *microenvironment.

Our results of a preserved cytokine secretion function of monocyte-derived macrophages in older volunteers confirm a report that compared macrophages only from women [34], but are in contrast to prior studies [16,35]. However, these divergent studies analyzed cells from remarkably older people than in our study, suggesting that monocyte functionality may decrease during further ageing. In elderly persons with a mean age 88 years, monocyte-derived macrophages were reported to display an imbalanced production of cytokines, with higher IL1β and IL6 at steady state as well as lower IL1β and higher IL6 and IL10 secretion upon stimulation [16]. Similarly, higher TNF, IL1β and IL6 levels were reported in macrophages from old (mean age 80 years) compared to young (mean age 27 years) subjects [35].

## Conclusions

Our study indicates that the circulating monocyte pool dynamically changes during ageing in humans. The expansion of the non-classical CD14^+^CD16^+ ^subtype, alterations of surface protein and chemokine receptor expression as well as circulating monocyte-related chemokines likely allow the preservation of functionality for the monocyte pool throughout the majority of adulthood.

## List of abbreviations used in the manuscript

CCR: C-C motif chemokine receptor; CCL: C-C-motif chemokine; DC: dendritic cell; Gr1: myeloid cell marker; IFNγ: interferon gamma; IL: interleukin; MCP-1: monocyte chemoattractant protein-1; MIP: macrophage inflammatory protein; PBMC: peripheral blood mononuclear cells; TNFα: tumor necrosis factor alpha.

## Authors' contributions

SS and HWZ performed the experiments, collected data and analyzed data. MB provided experimental tools. FT and CT designed the study, analyzed data and wrote the manuscript. All authors read and approved the final manuscript.

## Supplementary Material

Additional file 1 **Fig. S1. Age-dependent changes of HLA-DR and chemokine receptor expression on monocyte subsets**. (A) HLA-DR expression was analyzed by FACS on each monocyte subset. Box plots demonstrate alterations of HLA-DR expression on either CD14^++^CD16^- ^(left) or CD14^+^CD16^+ ^(right) monocytes by displaying the mean fluorescent intensity (MFI) of HLA-DR for the different groups of young (<30 years, n = 37), middle-aged (30-50 years, n = 50) and old (>50 years, n = 43) healthy volunteers. *p < 0.05. Click here for file
