# Supplementary material for: Age-dependent alterations of monocyte subsets and monocyte-related chemokine pathways in healthy adults
Source: BMC Immunol. 2010 Jun 21;11:30. doi: 10.1186/1471-2172-11-30 (PMC2910032; doi:10.1186/1471-2172-11-30)
Supplement: Additional file 1 — Fig. S1. Age-dependent changes of HLA-DR and chemokine receptor expression on monocyte subsets. (A) HLA-DR expression was analyzed by FACS on each monocyte subset. Box plots demonstrate alterations of HLA-DR expression on either CD14++CD16- (left) or CD14+CD16+ (right) monocytes by displaying the mean fluorescent intensity (MFI) of HLA-DR for the different groups of young (<30 years, n = 37), middle-aged (30-50 years, n = 50) and old (>50 years, n = 43) healthy volunteers. *p < 0.05. [file 1471-2172-11-30-S1.PDF]

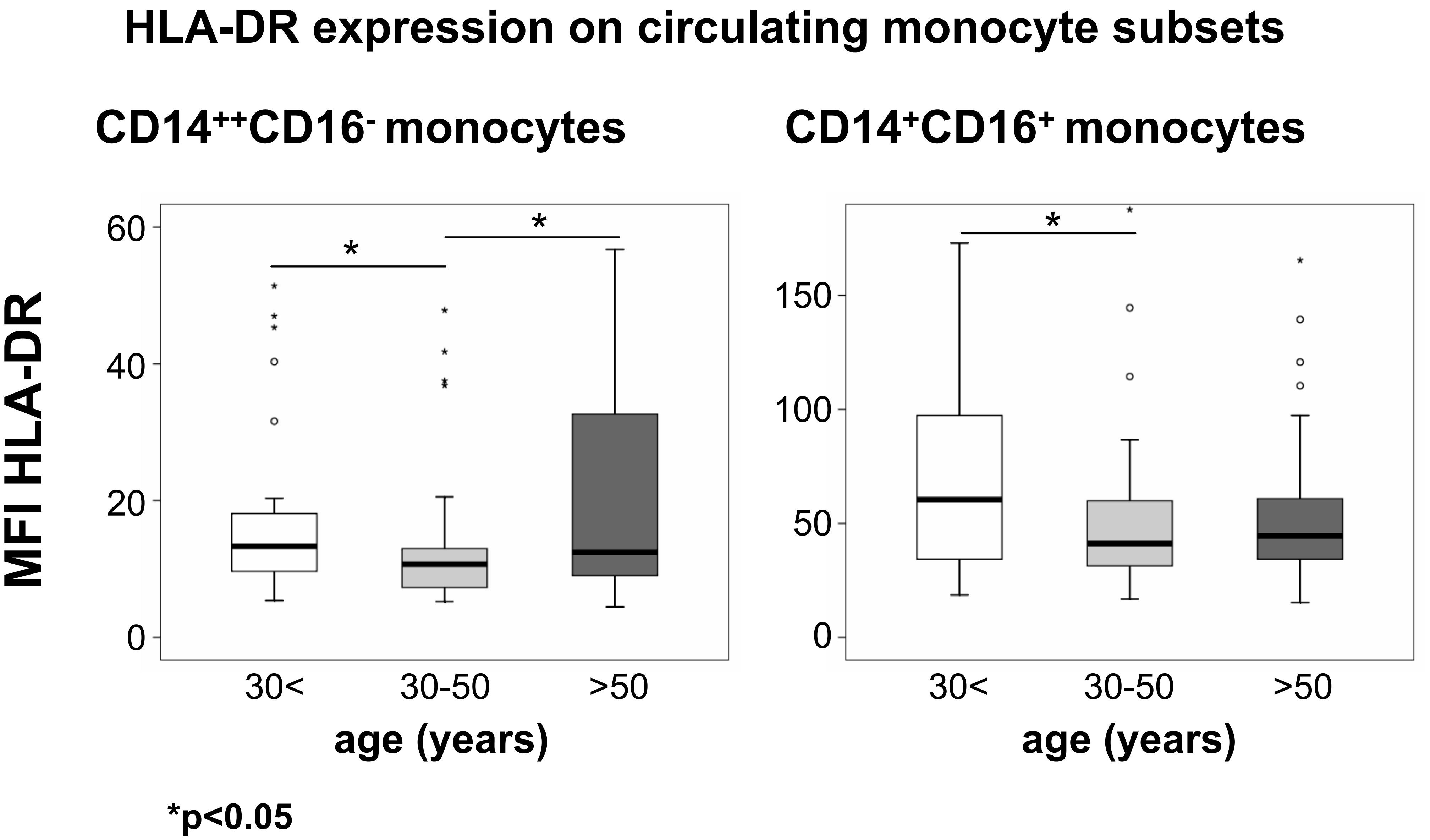
HLA-DR expression on circulating monocyte subsets
CD14++CD16- monocytes
CD14+CD16+ monocytes
MFI HLA-DR
0
20
40
60
0
50
100
150
*
*
*
30<
30-50
>50
30<
30-50
>50
age (years)
age (years)
*p<0.05
